# Impact of Intranasal Administration of Ayurveda Medicine in Apparently Healthy Individuals on Neurophysiological Variables and Functional Connectivity Using Functional Magnetic Resonance Imaging: Protocol for an Exploratory Randomized Controlled Trial

**DOI:** 10.2196/67132

**Published:** 2026-01-09

**Authors:** Devi R Nair, Sandya C J, Sophia Jameela, Remya E, Srikanth Moorthy, Harilal Parasuram, Shruti Khanduri, Bhogavalli Chandrasekhara Rao, Narayanam Srikanth, Rabinarayan Acharya

**Affiliations:** 1 National Ayurveda Research Institute for Panchakarma Thrissur, Kerala India; 2 Department of Radiodiagnosis Amrita Institute of Medical Sciences Kochi, Kerala India; 3 Central Council for Research in Ayurvedic Sciences New Delhi India; 4 Amrita Advanced Centre for Epilepsy Amrita Institute of Medical Sciences Kochi, Kerala India

**Keywords:** nasya, healthy individuals, neurophysiology, functional magnetic resonance imaging, functional MRI, blood oxygen level–dependent, BOLD, intranasal

## Abstract

**Background:**

*Nasyakarma*, an Ayurveda nasal drug delivery system, is considered a potent therapeutic modality in *panchakarma* treatment. Uniquely, nasal drug delivery can bypass liver metabolism and the blood-brain barrier for faster drug delivery. Available studies on *nasya karma* focus mainly on its efficacy. This pioneering study aims to explore the mechanisms of *nasya karma* on brain function and neurophysiology, investigating its potential to modulate activity in specific brain regions and affect the functional connectivity between these regions using functional magnetic resonance imaging (fMRI).

**Objective:**

The study aims to map the neurophysiological response of the brain to *nasya karma* using blood oxygen level–dependent fMRI in both rest and task phases and assess the impact of *nasya karma* on quality of life, cognition, sleep, and psychological well-being in healthy volunteers.

**Methods:**

A total of 60 healthy volunteers in the age group of 20 to 40 years who fulfill the selection criteria will be recruited for this randomized controlled trial at the National Ayurveda Institute for Panchakarma, Cheruthuruthy, Kerala, India, and randomized in a 1:1 ratio to either the intervention group (n=30; group 1, receiving *nasya karma* with *anu taila*, an oil-based formulation manufactured using a paste and decoction of herbal medicines with oils as the base material, for a period of 14 days) or the control group (n=30; group 2, not receiving any intervention). The participants will undergo task-based and resting fMRI on day 1 (twice on day 1 before administration and 15 minutes after *nasya karma* for participants in group 1) and day 14 to map the neurophysiological response to *nasya karma* of the brain. A comprehensive neuroimaging protocol using structural magnetic resonance imaging and fMRI will be used in the study. The effect of *nasya karma* on sleep, psychological well-being, cognition, and quality of life will be assessed on the 1st and 30th days in both groups.

**Results:**

After the data collection process of this randomized controlled trial is completed, the study will go into the analysis stage, in which the collected data will be subjected to robust statistical analysis. The final results of the study are expected to be published in 2026.

**Conclusions:**

This study will investigate the neurophysiological mechanisms of *nasya karma* by examining clinical, neuropsychological, and neuroimaging variables to identify associated neural patterns to develop therapeutic protocols for many diseases. The findings will also provide evidence for future research supporting the use of *nasya karma* as a practical and noninvasive therapeutic modality for treating cerebrovascular, behavioral, and neurological disorders as indicated in Ayurveda. The study will propel innovative research focusing on the neural mechanisms responsible for the delivery of central nervous system therapeutics to the brain, thereby bypassing the blood-brain barrier and yielding favorable outcomes in central nervous system diseases.

**Trial Registration:**

Clinical Trials Registry – India CTRI/2023/06/054219; hhttps://ctri.nic.in/Clinicaltrials/pmaindet2.php?EncHid=ODQ1OTU=&Enc=&userName=

**International Registered Report Identifier (IRRID):**

DERR1-10.2196/67132

## Introduction

### Background

In Ayurveda, *nasya karma* is among the *panchakarma* procedures, a set of 5 therapeutic modalities for body purification involving nasal administration of medicine, and is regarded as a potent approach in health promotive, preventive, and therapeutic applications. The therapeutic applications of *nasya karma* span a wide clinical spectrum, addressing conditions such as chronic sinusitis, cervical spondylosis, and various neurological and psychiatric disorders. *Nasya* *karma* is the only procedure that uses the nasal route for medicine administration to deliver therapeutic agents directly to the central nervous system (CNS), bypassing the digestive tract and potentially enhancing the efficacy of treatment owing to its faster absorption.

Intranasal drug administration has gained considerable traction for many decades as a noninvasive route for delivering medications targeting local, systemic, and CNS effects. The leaky intercellular junctions of the nasal mucosa and the extensive vascularity of the lamina propria and nasal mucosa facilitate optimal drug absorption, positioning intranasal delivery as a highly effective and versatile method for a wide range of therapeutic applications [1,2]. In the context of neurological disorders, intranasal drug delivery is advantageous due to its ability to achieve high drug levels in the brain, providing a direct pathway that bypasses the blood-brain barrier (BBB) more effectively than traditional parenteral and oral routes, especially considering that the anatomical features allow for rapid drug absorption, a noninvasive administration method, and the avoidance of hepatic first-pass metabolism, leading to faster therapeutic effects [3]. Direct absorption of molecules through the trigeminal and olfactory pathways provides a direct route to the brain, resulting in a favorable pharmacokinetic and pharmacodynamic profile by bypassing major physiological barriers such as the BBB and the blood–cerebrospinal fluid barrier [4].

The unique relationship that exists between nasal cavity and cranial cavity tissues makes intranasal drug delivery to the brain more feasible and effective. Intranasal delivery provides drugs with short channels to bypass the BBB, greatly enhancing the therapeutic effect on the neurological disease spectrum [5].

*Panchakarma* encompasses a comprehensive set of Ayurvedic detoxification procedures, including *vamana* (emesis), *virechana* (purgation), *nasya* (nasal administration of herbal oils or powders), *basti* or *vasti* (herbal enema), and *raktamokshana* (bloodletting) [6]. *Panchakarma* is a mainstay of Ayurveda clinical practice where the physician can choose any of the 5 sets of therapeutic modalities to bring about a homeostatic equilibrium in the body tailored to each individual depending on the intended purpose of use (promotive, preventive, therapeutic, or rehabilitative), body constitution, *dosha* (the body humors or functional principles that govern physiological and psychological processes), health or disease status, age, and fitness to undergo the *panchakarma* procedure.

*Anu taila* [7] is an oil-based formulation manufactured using a paste and decoction of herbal medicines with oils as the base material. The ingredients are combined in specific proportions as highlighted in classic Ayurvedic texts and subjected to a unique heating process to meet certain pharmaceutical parameters, ensuring the retention of both water-soluble and lipid-soluble principles at optimal levels. *Anu taila* can be likened to liposomes, where nanoparticles comprise lipid bilayer membranes surrounding an aqueous interior. The higher viscosity of this formulation increases contact time between the drug and the nasal mucosa, thereby enhancing permeation, and the viscosity may influence mucociliary clearance, potentially reducing it and, thus, prolonging the formulation’s retention time in the nasal cavity, which can lead to increased drug absorption [8,9].

Despite its extensive use as a therapeutic modality in clinical practice across a wide range of conditions and in healthy individuals to maintain health and homeostasis, the exact mechanism of action of *nasya karma* remains obscure and mysterious, primarily due to a lack of physiological or mechanistic studies in this area. Given the paucity of research on the neurophysiological mechanisms underlying *nasya*, this study aims to use functional magnetic resonance imaging (fMRI) to investigate its potential to modulate activity in specific brain regions and affect the functional connectivity between these regions. Understanding these neurophysiological correlates is crucial as it will elucidate whether *nasya karma* exerts targeted effects or has more widespread, nonspecific impacts on the brain’s intrinsic functioning. This knowledge will benefit stakeholders by providing a scientific basis for conceptualizing future research, potentially leading to optimized therapeutic protocols, improved clinical outcomes, and enhanced integration of *nasya karma* into modern medical practice. By establishing clear spatial and temporal patterns of brain activity associated with *nasya karma*, this study could pave the way for more precise and effective applications of this traditional Ayurvedic practice in contemporary health care. This may also propel innovative research focusing on the neural mechanisms responsible for the delivery of CNS therapeutics to the brain, thereby bypassing the BBB and yielding favorable outcomes in CNS diseases.

Although *nasya karma* is extensively used for neurological and systemic health benefits, no studies have elucidated its neurophysiological mechanisms of action, specific brain region targets, or the connectivity changes underlying its effects. This knowledge gap prevents scientific validation and optimized therapeutic application of *nasya karma* in clinical neuroscience. This study addresses this gap by using fMRI to characterize the brain’s response to *nasya karma*, thereby providing mechanistic insights essential for evidence-based integration of this ancient intervention into modern CNS therapeutics.

### Objectives

The primary objective of this study is to investigate the neurophysiological activity of the brain induced by the *nasya karma* procedure using blood oxygen level-dependent (BOLD) fMRI to elucidate the comprehensive neural response to *nasya karma* both in the rest and task phases. The secondary objective is to examine the impact of the *marsha nasya karma* (Ayurveda nasal drug delivery using a medicated or nonmedicated lipid medium) procedure on health parameters in apparently healthy volunteers, such as quality of life; cognition; sleep; and psychological well-being through anxiety, stress, and cognitive health.

The research hypothesis for this study is that the administration of *marsha nasya karma* with *anu taila* will increase the activation of different brain areas and enhance functional connectivity in different neuronal networks compared to baseline.

## Methods

### Ethical Considerations

The study will strictly adhere to the principles outlined in the Declaration of Helsinki (World Medical Association; 2013). Before enrollment in this trial, all participants will be required to provide informed consent by signing a consent form. The protocol has undergone rigorous ethical review and received approval from the institutional ethics committee of the National Ayurveda Research Institute for Panchakarma (NARIP), Cheruthuruthy, Thrissur, Kerala, Peripheral Institute of CCRAS, Ministry of Ayush, Government of India and Scientific Review Board, Amrita Institute of Medical Sciences, Kochi (F.No.8/16/2023/NARIP/Tech meeting/2508 dated March 31, 2023), and the study is registered with the Clinical Trials Registry – India. The participants will be given incidental support charges of INR 100 (US $1.11) per visit as participant compensation. The trial is covered by clinical trial insurance for any incidents, and the participants will receive appropriate compensation if these arise.

Protocol amendments, if any are needed in the course of the trial, will be communicated to the institutional ethics committees at Amrita Institute of Medical Sciences, Kochi, and NARIP, Cheruthuruthy, India. Personal information about potential and enrolled participants will be collected, shared, and maintained in a confidential manner before, during, and after the trial unless subjected to judiciary (ie, court-related) disputes. Access to trial data will be granted only to the research team involved at NARIP and the Amrita Institute of Medical Sciences (AIMS). There will be no public access to the trial dataset. Dissemination policy includes publication of study findings in peer-reviewed indexed international journals.

### Eligibility Criteria

Apparently healthy volunteers aged 20 to 40 years who are physically independent, ambulatory, and right-handed; have a BMI of 18.5 to 24.9 kg/m^2^; and are willing to participate and provide written informed consent will be included in the study. Only those who agree to abstain from beverages such as coffee, tea, energy drinks, or other stimulants beginning 12 hours before undergoing fMRI will be considered for recruitment. The healthy status of volunteers will be confirmed through the absence of any active or chronic disease, as determined through a comprehensive medical evaluation that will include a detailed medical history, complete physical examination (including vital signs), 12-lead electrocardiogram, hematology, blood chemistry, serology, psychological assessment, and urinalysis, among other tests.

Individuals with any disease or symptom, with a history of psychiatric disorders as assessed using the Mini-International Neuropsychiatric Interview, with psychosomatic disorders, who used psychotropic medications known to interfere with the dopaminergic system or sedatives within the previous 6 months, with a family history of psychiatric diseases, with past or current abuse of psychoactive drugs, or who use alcohol or smoke will be excluded. Individuals contraindicated for magnetic resonance imaging (MRI), such as those with medical implants, retained wires (eg, temporary pacing), an increased risk of myocardial infarction or other cardiac problems, known pregnancy, nausea (due to aspiration risk), metallic dental work, cochlear implants, body jewelry, and other metallic foreign objects implanted in the body will not be eligible either. Individuals with cognitive impairment, women in the menstrual phase, those with intracranial abnormalities that could adversely affect the interpretation of brain scans, and those who self-identify as claustrophobic are also not eligible for participation in the study. In addition to this, participants deemed unfit for *nasya karma* therapy [10], such as those with fever, rhinitis, cough, or other inflammatory conditions as indicated in the Ayurveda treatises, will be excluded.

### Interventions

The participants in the intervention group will be administered *nasya karma* (nasal instillation) with *anu taila* at a dose of 3 mL per nostril for 7 days (on every alternate day for 14 days) as indicated in the Ayurveda treatises for healthy individuals. Before *nasya*, local oleation and sudation of the head region will be conducted for approximately 10 minutes. Following the *nasya karma* procedure, participants will inhale medicated smoke and rinse their mouths with lukewarm water. The control group, consisting also of healthy individuals, will not undergo any interventions to facilitate assessment of the specific effects attributed to *nasya karma* in the intervention group when compared to baseline measures in healthy individuals.

*Nasya karma* will be administered by the investigator at the hospital’s *panchakarma* unit except on assessment days, when it will be conducted at AIMS. Each participant will be closely monitored for 30 minutes following the procedure to observe any immediate effects or reactions. Vital signs such as heart rate, blood pressure, and respiratory rate will be monitored and recorded before and after every session. Participants reporting any adverse symptoms experienced after each session will be recorded through a standardized format designed to capture a wide range of potential side effects, including nasal irritation, headache, dizziness, nausea, or any other discomfort. Any serious adverse event will be reported immediately to the institutional ethics committee, and a causality assessment will also be conducted. Appropriate care will be provided for all adverse events requiring medical intervention, and they will be followed up on until resolution or stabilization.

If a participant experiences significant adverse effects, such as severe nasal irritation, fever, sinusitis, or any other serious discomfort that impacts their well-being, the intervention will be immediately halted, and the participant will be withdrawn. Discontinuation will also occur in cases of noncompliance with the treatment regimen, if the participant wishes to withdraw for any reason, or if the participant meets any of the exclusion criteria during the study. As the trial involves healthy volunteers, administration of concomitant medication is prohibited during the trial period of 30 days.

**Figure 1:**
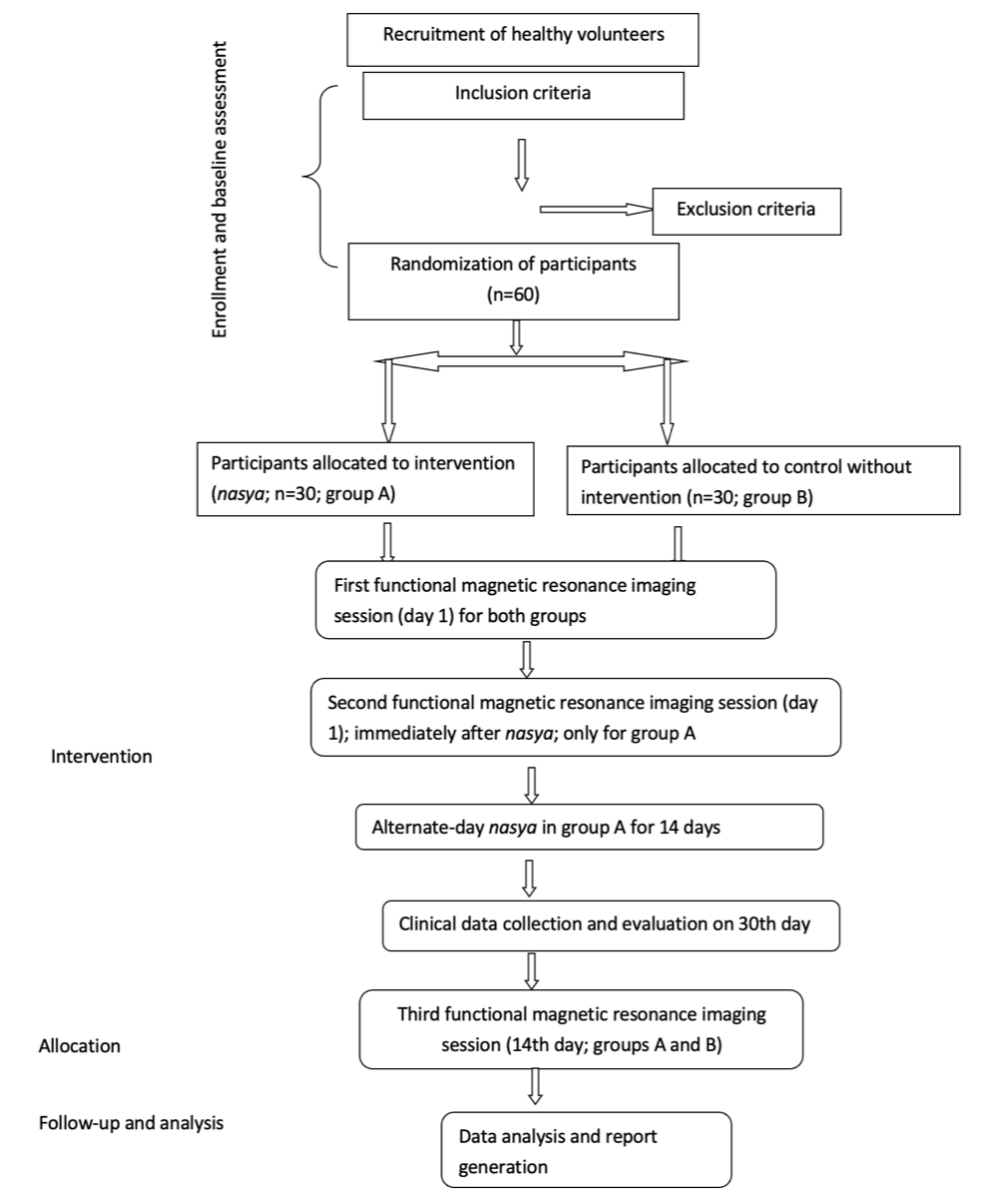
Schematic diagram of study plan.

This study is structured as an exploratory randomized controlled clinical trial, with participants allocated in a 1:1 ratio to the intervention and control groups, matched for age and sex to comprehensively assess and evaluate the neurophysiological and holistic health effects of the *marsha nasya karma* procedure in comparison to a control group (Figure 1). Settings of the study include NARIP and AIMS.

### Study Setting

Participants will be screened and enrolled at the NARIP in Cheruthuruthy, Thrissur, an institute under the Central Council for Research in Ayurvedic Sciences, Ministry of Ayush, government of India. The institute is well equipped for comprehensive Ayurvedic assessments and interventions, ensuring a high standard of participant care and protocol adherence during the *nasya karma* procedure. fMRI assessments will be carried out at AIMS, which is equipped with state-of-the-art imaging technology and experienced radiologists, providing the advanced neuroimaging capabilities necessary for detailed BOLD fMRI studies.

### Sample Size

As this is designed as an exploratory study aiming to gather preliminary data on the neurophysiological effects of the *nasya karma* procedure and its impact on various health parameters, a sample size of 60 (30 per group) is considered appropriate to achieve the study objectives and provide valuable insights that will inform the design of future, larger-scale clinical trials. Feasibility is a critical consideration as recruiting a larger sample of participants willing to undergo both the *nasya karma* procedure and twice-repeated fMRI assessments may be challenging, particularly among healthy volunteers. However, the expected variation in response to the interventions is likely to be lower than that in a patient population, which will allow for meaningful conclusions to be drawn from a smaller sample size. Similarly, a sample size of 60 balances the need for preliminary data with the practical constraints of the logistical and financial resources of the study.

### Randomization and Allocation

Eligible participants in this study will undergo random allocation to either the intervention or control group using a computer-generated random sequence prepared by an independent statistician, ensuring an unbiased approach to treatment assignment. Each random sequence number will be securely sealed in opaque envelopes, with the enrollment ID on the outside. Before enrollment, participants will open these envelopes to determine their allocated group to minimize bias.

### Primary Outcome Measure

The primary outcome measure for this study is the BOLD signal detected via fMRI in specific brain regions that are associated with motor, sensory, memory, and cognitive circuits elicited during both the rest and task phases. A 3-Tesla MRI scanner will be used to assess the brain’s response to different stimuli at multiple time points: before the *nasya karma* procedure, immediately before the *nasya karma* procedure, immediately after (within 15 min) the *nasya karma* procedure, and on the 14th day of the *nasya karma* therapy.

### Secondary Outcome Measures

These include the therapeutic effects of *marsha nasya karma* on cognitive function assessed via the Montreal Cognitive Assessment scale [11], perceived stress assessed using the Perceived Stress Scale [12], anxiety assessed using the Hamilton Anxiety Rating Scale [13], and health-related quality of life assessed using the 36-Item Short Form Health Survey [14]. The secondary outcomes will be assessed before the intervention and on the 1st and 30th days following the *nasya karma* therapy.

### Detailed Methodology of the Primary Outcome Measure

#### Overview

The primary outcome measure encompasses 2 key components. The first is the assessment of the immediate neurophysiological response elicited by *nasya karma* during both the resting and task phases using MRI. The second component is the evaluation of the neurophysiological response elicited by *nasya karma* after 14 days (following 7 days of nasya) during both the resting and task phases using MRI. Functional neuroimages will be collected using a 3-Tesla scanner (Discovery MR750w; GE HealthCare). Resting state fMRI data will include 3D T1-weighted structural imaging with a fast-spin echo sequence and BOLD imaging with a spin echo, gradient echo, and echo-planar imaging sequence for 40 minutes, covering the entire brain. The parameters for 3D-T1 imaging will include a slice thickness of 1 mm; a repetition time and echo time of 8.5 milliseconds and 3.2 milliseconds, respectively; a field of view of 256 × 256 × 142 mm^3^; a flip angle of 12°; a matrix of 256 × 256; an in-plane resolution of 1 × 1 × 1 mm^3^; and 142 layers. The parameters for BOLD imaging will include a slice thickness of 3.2 mm; a repetition time and echo time of 3000 milliseconds and 30 milliseconds, respectively; a flip angle of 80°; a matrix of 64 × 64; a field of view of 256 × 256 × 142 mm^3^; 35 layers; and 150 time points.

#### Preparatory Phase

Participants will undergo a robust screening interview to ensure understanding and absence of contraindications for MRI scanning. Before the task phase fMRI, participants will be thoroughly informed and trained. Communication with the operator will be maintained throughout the scan. Incidental findings requiring medical intervention will be disclosed, and participant comfort will be ensured during scanning. All scans will be reviewed by a qualified radiologist, and any clinically significant incidental findings will be communicated confidentially to the participant along with appropriate referral advice for further evaluation and management as per institutional protocols. Verbal communication will be limited due to MRI noise, and the participants will use a 4-button fiber-optic response box positioned for use with their right hand to communicate and record behavioral responses during tasks. To mitigate the effects of scanner noise, participants will wear earplugs. Head motion will be minimized using foam pads and a headband to ensure stable positioning throughout the MRI scans. Metallic items will be removed, and participants will wear MRI-compatible attire. Participants in both the trial and control groups will be directed to avoid heavy meals within 2 hours before and 1 hour after *nasya karma*. Both groups will refrain from consuming coffee, tea, or caffeinated drinks for 12 hours preceding the fMRI. In contrast, the control group will undergo resting state mapping on both the 1st and 14th days, whereas the participants in the intervention group will undergo the *nasya karma* procedure lasting approximately 30 to 45 minutes, after which participants will proceed to the MRI room. Participants in the control group will proceed directly to the MRI without any intervention.

#### Data Collection

##### Resting Phase fMRI

In this study, a block design paradigm will be used during task phases before and after *marsha nasya karma*, where stimuli are presented continuously within specific blocks alternated with rest periods to elicit and distinguish brain responses. Resting state fMRI, on the other hand, captures spontaneous brain activity without external stimuli, allowing for the correlation of signal fluctuations among functionally related brain regions. Panbrain activation mapping will be conducted during the resting phase before and immediately after *nasya karma* therapy (within 15 min) in the trial group. fMRI will be conducted before the *nasya karma* procedure and then again after the complete procedure is over. Participants in the control group will proceed directly to fMRI. During scanning, participants will be instructed to remain still with their eyes closed for 15 minutes to avoid motion artifacts. Any head movement will necessitate repeat mapping. The resting state fMRI paradigm will map spontaneous brain activity across the visual, auditory, motor (left and right), memory, and language domains before and after *marsha nasya karma*.

##### Task-Based fMRI

###### Overview

The task paradigms in this study are designed to effectively stimulate relevant brain areas while ensuring that participant performance remains accurate. Rest blocks are strategically interspersed between active blocks to establish baseline fMRI signals and allow participants to rest. Each domain collects sufficient data through a structured design featuring at least 4 blocks per task. Task durations are optimized to maintain participant concentration without inducing fatigue; breaks are included if needed due to head movements, ensuring image consistency.

###### Visual fMRI

The study will use a checkerboard method with 8-Hz flickering alternating with 30-second rest blocks in a sequence of 1 rest cycle plus 4 flickering cycles (B, AB, ABAB, and AB). Participants receive prior education and training to understand their tasks: viewing a flickering checkerboard followed by a fixation cross during rest periods. The paradigm runs for 4 minutes and 30 seconds, minimizing eye movements while allowing for effective scene categorization.

###### Auditory fMRI

Auditory tones (1000 and 2000 Hz; ratio of 4:1) alternate with 20-second *veena* sounds in a sequence of ABABABABABAB. Participants, relaxed with their eyes closed, hear alternating tones during rest periods, with more frequent standard tones. Each auditory block is followed by a 20-second *veena* sound. The paradigm duration is 4 minutes.

###### Motor fMRI (Right and Left)

Right and left motor areas are separately mapped using hand movements. Participants perform finger movements indicated by a flashing green dot on the screen alternating with 30-second rest blocks (1 off plus 4 on cycles [BABABABAB]). They synchronize opening and closing fist movements with the dot, followed by rest periods focusing on a fixation cross. Each motor task runs for 4 minutes and 30 seconds per hand.

###### Memory fMRI

The working memory attention task lasts 6 minutes and 45 seconds, presenting numbers for 0.5 seconds with 2.5 seconds of a black screen. Blocks of 51 seconds feature numbers with 30-second rest periods in between. Participants press a button when presented with identical numbers, with task initiation and closure including 15-second rest blocks. This paradigm effectively tests working memory under fMRI conditions.

###### Principles Underlying Assessment of Brain Response to Nasya Karma

The response to *nasya karma* will be assessed considering 3 principles. The first principle is that neural activity correlates with changes in local blood oxygen levels. The second principle is that oxygenated blood exhibits different magnetic properties compared to deoxygenated blood. The third principle is that alterations in the ratio of oxygenated to deoxygenated blood, known as the hemodynamic response function, can be inferred using fMRI through the measurement of the BOLD response.

#### Blinding

In this study, participant blinding is not feasible as the intervention involves a procedural administration (*nasya karma*) where participants are aware of undergoing the procedure and the study primarily aims to evaluate the procedural effect rather than an isolated pharmacological effect. Assessor blinding will also not be implemented as the primary outcome measure is fMRI, an objective instrumental assessment that is not subject to assessor interpretation bias during data acquisition.

#### Data Management

Data pertaining to the study, including screening data, recruitment data, baseline information, daily *nasya karma* procedure assessments, laboratory test reports, and secondary outcome measures assessed using scales, will be comprehensively documented in case report forms within 24 hours, and electronic case report forms will be completed within 7 days. Only the study personnel, individuals delegated with the task of data entry, and the statisticians will have access to the data. The imaging data generated from fMRI will be stored in hard disks at AIMS and will undergo further analysis after completion of the study. All study documents will be securely stored for 5 years following study completion.

#### Statistical Analysis

##### Overview

The modified intention-to-treat principle will guide the analysis for all outcomes within the full analysis set. Quantitative data will be summarized using mean, SD, minimum, maximum, and median values. Enumeration data will be presented as frequency and the corresponding percentage. Intragroup comparisons will be conducted using the Student paired *t* test or Wilcoxon signed rank test for continuous variables and the chi-square test for categorical variables. Intergroup comparisons will be conducted using the independent-sample *t* test or the Mann-Whitney *U* test. Significance thresholds will be set at *P*<.05 for all 2-tailed tests.

##### fMRI Data Analysis

The fMRI data will undergo preprocessing using either the Data Processing and Analysis for Brain Imaging or CONN toolboxes, which will encompass essential steps such as slice timing correction, realignment for motion correction, regression to remove confounding effects, normalization to a standard template, smoothing to enhance signal-to-noise ratio, detrending to remove low-frequency drifts, and filtering to focus on relevant frequency bands, aligning with established practices in the field. After data preprocessing, amplitude of low-frequency fluctuation, functional connectivity, and large-scale functional brain network analysis will be conducted to investigate the difference in neurophysiological responses between the groups. The results will be interpreted in the context of the experimental paradigm to elucidate neural correlates of the studied phenomena, providing insights into the effects of *nasya karma* on brain function. Voxels exhibiting expected time-course correlations (30-second periods) will receive high activation scores, whereas those showing no correlation or deactivation will receive lower or negative scores, respectively. MRI data analysis will use general linear models via statistical parametric mapping (SPM). fMRI data will be processed using MATLAB 2023 (MathWorks Inc).

Resting phase MRI data will be analyzed using software tools such as SPM, the CONN toolbox, and the Functional Magnetic Resonance Imaging of the Brain Software Library (Analysis Group), whereas task phase fMRI will specifically use SPM. Given the focus on group data in this study, complex patterns of brain activation will be interpreted collectively rather than individually. For voxel-based analysis, a significance threshold of *P*<.01 at the voxel level and *P*<.05 corrected for false discovery rate (FDR) at the cluster level will be applied. Connectome-based analysis will use a threshold of *P*<.05 corrected for FDR. Additionally, Pearson correlation analysis will be performed to explore potential associations between clinical outcomes and fMRI findings, examining changes in brain activity following different interventions.

##### Multiple-Comparison Correction

For voxel-wise analyses, statistical inference will be performed with the following corrections. At the voxel level, uncorrected *P*<.001 will be used as an initial threshold. At the cluster level, familywise error (FWE) correction (*P*<.05) will be applied to control for multiple comparisons across the brain. In parallel, FDR correction will be applied where appropriate to control the expected proportion of false positives. For independent component analysis–based network analyses, dual regression outputs will be thresholded using FWE correction (*P*<.05). This combination ensures robust control of type I error.

##### Power Analysis

A priori power analysis was conducted using G*Power (version 3.1) and the fMRIPower toolbox. On the basis of pilot data and prior studies of similar paradigms, an expected effect size of Cohen *d*=0.6 (moderate) was used. For a voxel-level threshold of *P*<.001, uncorrected and cluster-level FWE of *P*<.05, and a sample size of 30 per group, the analysis achieves >80% power to detect activation differences in relevant cortical regions. This power analysis informed our sample size and statistical thresholds.

##### Correlation of Clinical Outcomes and fMRI Data

Associations between clinical measures and fMRI-derived metrics (amplitude of low-frequency fluctuation, functional connectivity, and network strength) will be assessed using partial Pearson correlations controlling for age and sex. Given the multiple regions and measures assessed, the Benjamini-Hochberg FDR correction (*q*<0.05) will be applied to adjust for multiple testing across correlations. In addition, exploratory Spearman correlations will be performed to confirm the robustness of the observed associations.

#### Protocol Adherence Monitoring

In this study, all *nasya karma* procedures will be administered at the NARIP by trained investigators on alternate days. Participants will not perform *nasya karma* at home. This approach ensures direct procedural supervision, standardized administration, and immediate monitoring for any adverse effects or participant discomfort. Direct assessment by the investigator at each session enhances protocol adherence, fidelity, and the overall robustness of study execution, eliminating the variability associated with home-based administration and, thereby, strengthening the internal validity and reliability of the study findings as in Table 1.

**Table 1:** Gantt chart.

		Week 0	Baseline		Intervention period (1st-13th day)	Follow-up	
			1st day	1st day (immediately after *nasya*)		14th day	30th day
Participant screening, including laboratory tests		✓					
Informed consent		✓					
Randomization		✓					
Intervention: *nasya karma* (group A)			✓		✓		
Control (group B): without *nasya karma*							
**fMRI** ^a^ **scan**							
	Group A		✓	✓		✓	
	Group B		✓			✓	
**Clinical assessment**							
	Group A						✓
	Group B						✓

^a^fMRI: functional magnetic resonance imaging.

## Results

After the data collection process of this randomized controlled trial is completed, the study will go into the analysis stage, in which the collected data will be subjected to robust statistical analysis. The final results of the study are expected to be published in 2026.

## Discussion

### Anticipated Findings

This study is anticipated to provide preliminary evidence of the neurophysiological responses evoked by the Ayurvedic procedure of *nasya karma* (nasal instillation) on specific brain areas. The findings are expected to demonstrate distinct neuronal activation patterns within visual, auditory, motor, and cognitive domains both immediately (within 15 min) and after cumulative administration (14 days). This will be the first mechanistic exploration mapping the impact of *nasya karma* on brain regions using neuroimaging techniques, bridging traditional Ayurvedic concepts and objective scientific validation.

### Comparison to Prior Work

While numerous studies have established the efficacy of *nasya karma* in treating conditions such as sinusitis, headache, and neurological disorders, these works predominantly focus on clinical outcomes rather than mechanistic pathways. Previous pharmacological and anatomical studies have highlighted the naso-brain route as a potential noninvasive drug delivery pathway bypassing the BBB. However, the specific neural activation patterns evoked by *nasya karma* procedures remain unexplored in Ayurveda literature. Thus, this study complements existing clinical research by providing neurophysiological evidence to support Ayurveda’s theoretical claims, potentially validating traditional beliefs under modern neuroscientific paradigms.

### Strengths and Limitations

The study is a novel approach combining Ayurvedic procedures with neuroscientific imaging methods to generate mechanistic evidence. The mapping of immediate and cumulative neuronal responses that can inform both therapeutic and preventive medicine applications is the key highlight of the study. The study focuses on differential activation across brain domains, providing granular insights rather than generic findings.

Limitations include the small sample size (n=30 per group) due to feasibility constraints in recruiting healthy volunteers for radiological assessments, potentially affecting generalizability. Administration in the outpatient setting limits investigator control over adherence, introducing protocol compliance variability as yet another limitation. As an exploratory study, the design is not powered to evaluate clinical efficacy, focusing solely on mechanistic end points.

### Future Directions

Findings from this study will act as preliminary data for larger controlled mechanistic trials evaluating the *nasya karma* procedure for specific neurological conditions such as cognitive impairment, stroke, neurodegenerative disorders, and mood disorders. Optimization of dosage, formulation, and timing parameters for maximum neural impact is another future direction from the study. The study paves the way to translational neuroscience research into Ayurveda therapies as noninvasive approaches to modulate brain function and deliver CNS-targeted treatments via the nasal route.

### Dissemination Plan

The results of this research will be disseminated through both national and international peer-reviewed publications and summary briefs for Ayurveda practitioners and policymakers. This aims to integrate traditional procedures into evidence-based neurological practice and stimulate interdisciplinary research between Ayurveda and modern biomedical sciences.

### Conclusions

This study is expected to provide first-of-its-kind neurophysiological evidence supporting the mechanistic action of the *nasya karma* procedure in the brain. By bridging traditional Ayurveda concepts with modern neuroscience, it has the potential to pave the way for innovative interventions in neurological disease management and preventive health care.

## Appendix

Multimedia Appendix 1 Peer review report from the Project Evaluation Monitoring Committee, Central Council for Research in Ayurvedic Sciences, Ministry of AYUSH, Government of India.

## Abbreviations

AIMS: Amrita Institute of Medical Sciences
BBB: blood-brain barrier
BOLD: blood oxygen level–dependent
CNS: central nervous system
FDR: false discovery rate
fMRI: functional magnetic resonance imaging
FWE: familywise error
MRI: magnetic resonance imaging
NARIP: National Ayurveda Research Institute for Panchakarma
SPM: statistical parametric mapping
