# Supplementary material for: Impact of Intranasal Administration of Ayurveda Medicine in Apparently Healthy Individuals on Neurophysiological Variables and Functional Connectivity Using Functional Magnetic Resonance Imaging: Protocol for an Exploratory Randomized Controlled Trial
Source: JMIR Res Protoc. 2026 Jan 9;15:e67132. doi: 10.2196/67132 (PMC12831104; doi:10.2196/67132)
Supplement: Multimedia Appendix 1 [file resprot_v15i1e67132_app1.pdf]

केन्द्रीय आयुर्वेदीय विज्ञान अनुसंधान परिषद्
आयुष मन्त्रालय, भारत सरकार
जवाहर लाल नेहरू भारतीय चिकित्सा एवं होम्योपैथी अनुसंधान भवन
61-65, सांस्थानिक क्षेत्र, सम्मुख 'डी' ब्लाक, जनकपुरी, नई दिल्ली-110058
CENTRAL COUNCIL FOR RESEARCH IN AYURVEDIC SCIENCES
Ministry of AYUSH, Govt. of India
Jawahar Lal Nehru Bhartiya Chikitsa Evam Homoeopathy Anusandhan Bhawan
61-65, Institutional Area, Opp. 'D' Block, Janakpuri, New Delhi-110058

Fax : 28520748
EPBX
28525852, 28520501
28522524, 28525831
28525862, 28525883
28525897

F. No. HQ-ADMN018/40/2022-ADMN /5725

Dated: 20 JAN 2023

Sub: Minutes of 3rd Meeting of Project Evaluation Monitoring Committee (PEMC) held on 12th January, 2023 at 10.00 am in hybrid mode at CCRAS Hqrs., New Delhi.

Sir/Madam,

I am directed to enclose herewith the Minutes of the 3rd Meeting of Project Evaluation Monitoring Committee (PEMC) held on 12th January, 2023 at 10.00 am in hybrid mode at CCRAS Hqrs., New Delhi for kind information.

Yours faithfully,

(Dr. Adarsh Kumar)
Assistant Director (Ay.)
For Director General

To,

1. **Prof. (Dr.) Rabinarayan Acharya, Director General,** CCRAS, New Delhi **Chairman**
2. **Dr. S. K. Benara,** Former Scientist F, ICMR-NIMS, New Delhi
3. **Dr. Atul Juneja,** Former Scientist E, ICMR-NIMS, New Delhi
4. **Mr. Raj Kumar,** Director (IFD), Ministry of Ayush, New Delhi.
5. **Dr. Sunita Garg,** Former Chief Scientist (Sci.- G)
6. **Prof. (Dr.) Suresh Kumar,** Dept. of Botany, Ramjas College, delhi
7. **Dr. Rajeev Sharma,** Former Director, PCIM, Gaziabad, U.P
8. **Dr. Sharad Srivastav,** Senior Principal Scientist and Head, Pharmacognosy Division, NBRI, Lucknow
9. **Prof. Yogender Pal Khasa,** Department of Microbiology, University of Delhi, Delhi
10. **Dr Sonia Khatter,** Professor(Microbiology), ESIC, Delhi
11. **Dr. Gian Singh,** Former Scientist 'F' and Head, NISCAIR, Delhi
12. **Dr. Virendra Singh Rana,** Principal Scientist, IARI
13. **Dr. S. K. Maulik,** Former professor, AIIMS, Delhi
14. **Dr. Sharad Wakode,** Prof. (Pharmaceutical Chemistry), DIPSAR, Delhi
15. **Dr.Galib,** Associate Professor, AIIA
16. **Prof. B.J.Patgiri,** (Dean Research), ITRA, Gujarat
17. **Prof. Anil Singh,** Department of Dravyaguna, IMS,BHU, Varanasi
18. **Dr.Sudipt Rath,** Associate Professor ,Dept. of Dravyguna
19. **Dr. M.M. Padhi,** Ex-DDG, CCRAS, Bhubaneswar

20. **Dr.Asit K. Panja,** Associate Professor, NIA, Jaipur
21. **Dr. Arun Gupta,** Professor Panchakarma, CBPACS, Delhi
22. **Prof. A.C.Kar,** Vikriti Vigyan, IMS, BHU,Varanasi
23. **Dr. N. Srikanth,** Deputy Director General, CCRAS, New Delhi **Member Secretary**

Copy to:

1. All Concerned Programme Officers/ Nodal Officers with request to process the files/e-files for necessary sanction ensuring the necessary compliance.
2. The dealing officer from Technical and Admn. Section with direction to process the file for each project separately through file concerned/e-office.
3. Directors/In-charges of CCRAS peripheral Institutes/Centres/Units for taking necessary action.
4. Ad. O (Projects) for necessary action related to sanction of the approval projects
5. Accounts Officer, CCRAS and Budget Section for record
6. Sr. PS to DG/PS to DDG, CCRAS, New Delhi.

-Sd-

For Director General

Central Council for Research in Ayurvedic Sciences

Minutes of the 3rd meeting of Project Evaluation Monitoring Committee (PEMC) held on 12th January, 2023 at 10.00 am in hybrid mode at committee room of CCRAS headquarters

The 3rd meeting of Project Evaluation Monitoring Committee (PEMC) was held on 12th January, 2023 at 10.00 am in hybrid mode at committee room of CCRAS headquarters, New Delhi under the chairmanship of Prof Vaidya Rabinarayan Acharya, Director General, CCRAS. The list of committee members and CCRAS officers who attended the meeting are annexed **at Annexure I.**

At the outset, Prof Vaidya Rabinarayan Acharya, chairman of the PEMC, welcomed all the members of the committee & participants and briefed about the agenda for the PEMC meeting. After that each Agenda item was placed and discussed in meeting in detail. After a thorough discussion, the committee unanimously decided and gave some common recommendations/ observations on technical and financial aspect of research projects followed by agenda wise observations. The recommendation/suggestions of the committee on each individual proposal discussed in the meeting are annexed at **Annexure –II**.

GENERAL RECOMMENDATION:

Technical

- Research question, hypothesis and rationale should be included in all clinical trial protocols and accordingly summary format may be revised.
- Insurance coverage should be mandatory in all clinical trials.
- Data Safety Monitoring Board (DSMB) may be constituted for multicenter, large scale studies for addressing the safety monitoring and risk assessment of study participants.
- Specific experts may be called for monitoring in future.
- ISC and other committee's Recommendation may be included in the agenda of PEMC meetings in future.
- In future there should be monitoring and review of each ongoing project.

Finance

- During purchase of equipments/instruments, codal formalities as per GFR 2017 has to be complied.
- Budget for furniture should not be included in the project budget.
- In the collaborative projects, ownership of all non-recurring assets, procured in the project, shall be mentioned in the MoU.
- The Expert/ Consultant may be engaged on per visit basis 'not more than Rs.3000/- per visit and a maximum of Rs.30000/- per month'.
- No laptops will be provided in the budget of project.

Agenda 3.1: Action Taken Report of 2nd PEMC meeting: Total **65** projects were approved in 2nd PEMC meeting out of which **45** projects have been sanctioned, **10** projects are in the

process of sanctioning, **09** project proposals have been sent to the PIs for revision and SFC/IFD concurrence is to be obtained for **01** project.

Agenda 3.2: Clinical Research: Total **14** Research Project Proposals including **01**Supplementary Agenda were placed before the committee. After detailed discussion, **13** proposals at agenda items no. **3.2.1 to 3.2.13** were approved by the committee with certain modification & suggestions and the proposal at supplementary agenda has been deferred **(Annexure –II from Pg no. 5 -12)**.

Agenda 3.3: Medicinal Plant Research: Total **10** Research Project Proposals were placed before the committee. After detailed discussion, **02 projects** at agenda no. **3.3.3 & 3.3.5** were approved by the committee; **04 projects** at agenda no. **3.3.1, 3.3.2, 3.3.4 & 3.3.6** were approved with certain modification & suggestions and **04 projects** from agenda no. **3.3.7 to 3.3.10** were approved by the committee in principle for which physical meeting with PIs is to be convened **(Annexure –II from Pg no. 12-14)**.

Agenda 3.4: Drug Standardization Research: Total **07** Research Project Proposals including **04** Supplementary Agendas (S1 to S4) were placed before the committee. After detailed discussion, **02 projects** at agenda no. **3.4.1 & 3.4.3** were approved by the committee; **05 projects** at agenda no. **3.4.2, S1, S2, S3 & S4** were approved by the committee with certain modification & suggestions **(Annexure –II from Pg no. 14-16)**.

Agenda 3.5: Pharmaceutical Research: Total **02** Research Project Proposals were placed before the committee. After detailed discussion, **both the projects** at agenda no. **3.5.1 & 3.5.2** were approved by the committee with certain modification & suggestions **(Annexure – II from Pg no. 16)**.

Agenda 3.6: Pharmacology Research: Total **14** Research Project Proposals were placed before the committee at agenda no. **3.6.1 to 3.6.14.** After detailed discussion, **13 projects** were approved by the committee with certain modification & suggestions and **01 project** at agenda no**. 3.6.8** was deferred by the committee **(Annexure –II from Pg no. 16-21)**.

Agenda 3.7: Literary and Fundamental Research: Total **04** Research Project Proposals were placed before the committee. After detailed discussion, **01** proposal at agenda no. **3.7.2** was approved by the committee and **03 projects** at agenda no. **3.7.1, 3.7.3 and 3.7.4** were approved by the committee with certain modification and suggestions **(Annexure –II from Pg no. 21-22)**.

The Meeting ended with vote of thanks.

***

Annexure –I

List of Participants attended the 3rd Meeting of Project Evaluation & Monitoring Committee (PEMC)

A. Members who attended the meeting

1.	**Prof. Rabinarayan Acharya ,** Director General ,CCRAS ,New Delhi	Chairman
2.	**Dr. S.K. Banera** Former, Scientist F, ICMR-NIMS, New Delhi	Member
3.	**Dr.Atul Juneja,** Former Scientist E	Member
4.	**Mr.Raj Kumar,** Director (IFD), Ministry of Ayush, New Delhi	Member
5.	**Dr. Arun Gupta,** Professor Panchakarma,CBPACS, Delhi	Member
6.	**Dr. Sunita Garg,** Former Chief Scientist (Sci-G)	Member
7.	**Prof. Dr. Suresh Kumar,** Department of Botany, Ramjas College, University Enclave, Delhi-110007, India	Member
8.	**Dr. Rajeev Sharma,** Former Director, Pharmacopeial Commission of Indian Medicine(PCIM) Ramashray, Ghaziabad	Member
9.	**Dr. Sharad Srivastav,** Senior Principal Scientist and Head, Pharmacognosy Division,NBRI, Lucknow,U.P	Member
10.	**Prof. Yogender Pal Khasa,** Department of Microbiology, University of Delhi South Campus, New Delhi	Member
11.	**Dr. Gian Singh** Former Scientist 'F' and Head, Education Training and Translation Division, NISCAIR,EC 341, Maya Enclave, New Delhi	Member
12.	**Dr. Virendra Singh Rana,** Principal Scientist Natural Product Chemistry (Organic Chemistry) Indian Agricultural Research Institute, New Delhi (IARI)	Member
13.	**Dr. S. K. Maulik,** Former professor, AIIMS, H-1473, Second floor, CR Park, near CR Park police station, New Delhi	Member
14.	**Dr. Galib,** Associate Professor, AIIA,New Delhi	Member
15.	**Prof. Anil Singh,** Department of Dravyaguna, IMS,BHU ,Varanasi	Member
16.	**Dr. Asit K.Panja,** Associate Professor, NIA, Jaipur	Member
17.	**Prof. A.C. Kar,** Vikriti Vigyan, IMS, BHU, Varanasi	Member
18.	**Dr. M.M. Padhi,** Ex-DDG, CCRAS, Bhubaneswar	Member

B. CCRAS Officials who attended the meeting

1. Dr. Sanjay Kumar Y.R., AD (Pharmacology)
2. Dr. Ravinder Singh, AD (Chem.)
3. Dr. Adarsh Kumar, A.D. (Ay.)
4. Dr. B.C.S. Rao, A.D. (Ay.)
5. Dr. Arjun Singh, A.D. (Chem.)
6. Dr. Pratap Makhija, A.D. (Ay.)
7. Dr. Renu Makhija, A.D. (Path.)
8. Dr. B. Venkateshwarlu, A.D (Ay.)
9. Dr. Deepa Makhija, A.D (Ay.)
10. Dr. Sarada Ota, A.D (Ay.)
11. Sh.Dalip Jain, A.do(P &B)
12. Dr. Sunita, R.O .(Ay)

13. Dr. B.S. Sharma, R.O. (Ay)
14. Dr. Shruti Khanduri, R.O. (Ay)
15. Dr. V.K. Lavaniya, R.O. (Ay)
16. Dr. A.K. Jain, R.O. (Ay)
17. Dr. Renu Singh, R.O. (Ay)
18. Dr. Babita Yadav, R.O. (Ay)
19. Dr. Shiddamallayya, R.O. (Botany)
20. Dr. Rakesh Rana ,Statistical Officer
21. Dr. Lalita Sharma, R.O. (Ay)
22. Dr. Rakesh Narayanan , R.O. (Ay)
23. Dr. Sophia Jameela, R.O. (Ay)
24. Dr. Kalpana Kachare, R.O. (Ay)
25. Dr. Ashwathy Kutty, R.O. (Ay)
26. Dr. Bidhan Mahajon, R.O. (Ay)
27. Dr. Neha Dubey, R.O. (Ay)
28. Dr. Arunabh Tripathi , Statistical Officer
29. Dr. Amit Rai, RO (Ay.)
30. Dr. Pallavi S. Mundada, RO (Ay.)
31. Dr. Anagha Ranade, RO (Ay.)
32. Dr. Richa Singhal, Senior Statistical Assistant

Annexure –II

AGENDA WISE RECOMMENDATION

The detailed agenda wise comments and Recommendation are as below:

Agenda	Title of the project	PI from CCRAS	PI/CO-I from collaborating institute	Comments/minor revisions to be made/Recommendation
**Agenda 3.2. Clinical Research**				
3.2.1	A Randomized Controlled Study to Assess the Effect of Marsha Nasya Karma in Motor, Sensory, Memory and Cognitive Parameters elicited Through f – MRI in Apparently Healthy Individuals.	1.Dr.Devi R Nair, RO(Ay), NARIP cheruthuruthy	Dr. Srikanth Moorthy, Professor of Radiology, Amrita Institute of Medical Sciences, Kochi	1. EEG may be considered to be incorporated as a screening tool, if feasible<br>2. Use of beverages such as coffee, tea etc, should be limited prior to f-MRI<br>3. Imaging equipment shall be either a 1.5 T or 3 T, and may be finalised in consultation with the radiologist<br>4. Only one outcome may be kept as primary outcome and the remaining may be kept as secondary outcomes<br>5. In the selection criteria, the limits of scales that is used as assessment tools may also be incorporated, wherever applicable.<br>**Recommendation:** Approved with the suggestion that the final proposal may be submitted incorporating the aforementioned suggestions to the CCRAS Headquarters for final approval.
3.2.2	Efficacy and safety of *Punarnavadi Mandura* and *Dadimadi Ghrita* Vs standard care in Adolescents with Iron Deficiency Anaemia- A Randomized Active Controlled Trial	Dr Remya E, PhD(Ay), PGDYN Research Officer (Ay), NARIP, Cheruthuruthy		1. The title may be revised by incorporating the components of efficacy such as cognitive dysfunction, and functional gut wellbeing.<br>2. Standard regimen may be modified with addition of folic acid 5 mg also.<br>3. Medication adherence may be added as a secondary outcome measure<br>4. It shall be stated in the protocol that participants with < 80% compliance will be excluded from analysis.<br>**Recommendation:** Approved with the suggestion that the final proposal may be submitted incorporating the aforementioned suggestions to the CCRAS Headquarters for final approval.
3.2.3	To evaluate the effect of Ashwagandha treatment on sleep architecture in	Dr. Srinibas Sahoo, R.O. (Ay.), CARI, Bengaluru	Dr. PN Ravindra, Professor, NIMHANS, Bengaluru	1. The methodology may be revised as open label randomized controlled trial as the participants/ investigators cannot be blinded

	Anidra (chronic insomnia) and the associated mechanisms involved: A whole night polysomnography, Imaging, Biochemical and well-being assessment.			2. The outcome measures may be revised to address the parameters mentioned in the 05 objectives along with the tools for assessment<br>3. The interventions in the standard care/treatment as usual may be specified along with the dosage schedule<br>4. The relevance of inflammatory markers and their possible association with insomnia may be revisited and only specific markers may be kept as outcome measure and accordingly, budget may be revised.<br>5. The role and responsibilities of each investigator may be annexed in the proposal and also included in the MoU<br>6. Sample size may be revisited addressing the expected change in Ayurveda stand-alone care arm versus that of the treatment as usual arm<br>**Recommendation:** Approved with the suggestion that the final proposal may be submitted incorporating the aforementioned suggestions to the CCRAS Headquarters for final approval.
3.2.4	Efficacy, Safety, tolerability, and Neurobiological correlates of Ayurveda treatment as Adjunct to Established Maintenance Doses of Standard care, on negative and cognitive symptoms in Schizophrenia: A prospective randomized controlled trial	Dr. Tejaswini C, R.O. (Ay.), CARI, Bengaluru	Dr. Vijaya Kumar, Associate Professor Department of Psychiatry NIMHANS, Bengaluru	1. The budget for workstation and hard disk may be met up from the over-head charges<br>2. The term Post Hoc may be removed from the objectives and relevant outcomes may be finalized<br>3. The role and responsibilities of each investigator may be annexed in the proposal and accountability for the same may also be included in the MoU<br>4. Sample size may be finalized in consultation with statistician addressing CAINS<br>5. Method for assessment of medication adherence may be incorporated in the protocol<br>6. Therapeutic drug monitoring may not be feasible and medication adherence can be ensured through maintaining diary log for the same<br>7. Only relevant inflammatory markers may be kept and accordingly, budget shall be revised<br>**Recommendation:** Approved with the

				suggestion that the final proposal may be submitted incorporating the aforementioned suggestions to the CCRAS Headquarters for final approval.
3.2.5	Randomized control trial of "Anshumati Ksheer Paka" in hypertension induced left ventricular hypertrophy	Dr. Neha Dubey Research Officer (Ayurveda), CCRAS Hqrs	Dr. Puneet Gupta Assistant Professor (Cardio.), Safdarjung Hospital , New Delhi	1. Standard method of preparation of the trial intervention shall be mentioned in the protocol<br>2. Standard care shall be mentioned<br>3. The safety aspect of the drug may be highlighted and e-GFR may be included in the investigation<br>4. A measuring cup, spoon etc. shall be issued to participant to ensure SoPs<br>5. Patient shall be adequately trained to prepare the trial intervention<br>6. Alcohol use disorder and smoking may be included as exclusion criteria.<br>**Recommendation:** Approved with the suggestion that the final proposal may be submitted incorporating the aforementioned suggestions to the CCRAS Headquarters for final approval.
3.2.6	Efficacy of Avipattikara Churna and Chitrakadi Vati versus PPI (Proton Pump Inhibitor) in Decreasing Symptom Severity in Patients with Functional Dyspepsia- A Randomized Control Trial	Dr. Indu S, Research Officer, CARI, Bhubaneswar	Dr. Manas Kumar Panigrahi, AIIMS,BBSR	1. The study site may be kept as AIIMS, Bhubaneswar<br>2. The SRF (Ay) shall be posted at AIIMS for the execution of the study<br>3. The detailed budgetary breakup may be incorporated in the project<br>**Recommendation:** Approved with the suggestion that the final proposal may be submitted incorporating the aforementioned suggestions to the CCRAS Headquarters for final approval.
3.2.7	A randomized placebo control study to evaluate the effect of Ayush SR as adjunct to usual care for cardiac rehabilitation on biochemical, physical and psychological parameters after Coronary Artery Bypass Graft (CABG) surgery	Dr. Seema Jain, RO(Ay) Central Ayurveda Research Institute, Delhi	1.Dr. Shiv Kumar Choudhary, Department of Cardiothoracic & Vascular Surgery CT Centre, AIIMS, New Delhi<br>2. Dr Manoj Sahu, Associate Professor, CTVS, ICU	1. Justification for social worker may be added in the budget<br>2. If required, the study duration may be extended to enrol 220 participants fulfilling the selection criteria<br>3. Instead of furniture, medicine cabinet may be included for keeping trial intervention<br>**Recommendation:** Approved with the suggestion that the final proposal may be submitted incorporating the aforementioned suggestions to the CCRAS Headquarters for final approval.

			All India Institute of Medical Sciences, New Delhi	
3.2.8	Comparing the efficacy of Yashtimadhu Ghrita anal infiltration and Diltiazem topical application in the management of chronic anal fissure –An open-label randomized controlled trial	1. Dr. Hemanta Panigrahi, Central Ayurveda Research Institute, Delhi<br>2. Dr. Amit Rai, CCRAS Hqrs., New Delhi	Not applicable	1. Insurance component may be added in the budget, accordingly the budget may be revised.<br>2. Trial drugs : The justification shall include Preparation and standardization of the trial interventions and procurement of standard care<br>3. The translational value and IPR aspects may be revised<br>**Recommendation:** Approved with the suggestion that the final proposal may be submitted incorporating the aforementioned suggestions to the CCRAS Headquarters for final approval.
3.2.9	Clinical evaluation of Ayurvedic regimen (*Virechana Karma* followed by oral administration of Kankayana Vati, Kanchanara Guggulu and Kumaryasava) in the management of polycystic ovarian syndrome- a randomized controlled open label clinical trial	Dr Susmitha Otta RO(Ay), CARI, BBSR<br>Dr. Emy S Surendran RO(Ay), RARI, Trivandrum<br>Dr Binitha P RO(Ay), NARIP, Cheruthuruthy		1. The budget for laptop may be removed<br>2. The MTA requirement under the manpower head may be removed and data entry can be done by SRF. Budget may be revised accordingly<br>3. The Consultant Gynaecologist may be engaged on per visit basis not more than Rs.3000/- per visit and a maximum of Rs.30000/- per month.<br>4. The justification for the selection of 03 interventions may be provided in the proposal<br>**Recommendation:** Approved with the suggestion that the final proposal may be submitted incorporating the aforementioned suggestions to the CCRAS Headquarters for final approval.
3.2.10	"Efficacy of **Ayush Rasayana A & B** on Quality of Life of elderly population- A cluster randomized study" (under AMHCP –SCSP & THCRP)	09 institutes of THCRP<br>1. Dr. S. Doddamani, (CARI, Bangalore)<br>2. Dr. Ekta , (CARI, Guwahati)<br>3. Dr. M.M. Sharma, (RARI, Gwalior)<br>4. Dr. Vimal Tiwari		1. The Title of the project may be revised as "**Effectiveness** of **Ayush Rasayana A & B** on Quality of Life of elderly population- A cluster randomized study".<br>2. In primary and secondary objectives the word 'efficacy' may be replaced with 'effectiveness'.<br>3. Age of participants in inclusion criteria may be considered as ≥60 to ≤75 in place of ≥65 to ≤75 years<br>**Recommendation:** Approved with the suggestion that the final proposal may be submitted incorporating the aforementioned suggestions to the CCRAS Headquarters for final approval.

		,(RARI, Patna)<br>5. Dr. Vipin Sharma, (RARI, Jammu)<br>6. Dr. Srinivas Pitta, (ALRARI, Chennai)<br>7. Dr. Abhey Dev, (RARI, Portblair)<br>8. Dr. Ravate, (RARC, Agartala)<br>9. Dr. Nishanth, (RARI, Vijayawada)<br>12 institutes from AMHCP-SCSP<br>1. Parvathy G Nair (NARIP, Cheruthuruthy)<br>2. Dr. Gurucharan Bhuyan(CARI Bhubaneswar)<br>3. Dr. Ranjita Ekka (CARI, Kolkata)<br>4. Dr. Amrish (CARI ,Mumbai)<br>5. Dr Thejaswini C (CARI, Bangalore)<br>6. Dr. Sinimol. T. P (RARI Thiruvananthapuram)<br>7. Dr. Alok Kumar		

		Srivastava (RARI, Lucknow)<br>8. Dr. U. R. Sekhar Namburi (RARI, Nagpur)<br>9. Dr.Sariga .K.S (RARI, Gangtok)<br>10. Dr Gopesh Sharma (RARI, Jammu)<br>11. Dr Kavita Vyas (RARI, Mandi)<br>12.Dr.Parth dave (RARI, Ahmedabad)		
3.2.11	"Effectiveness of complex Ayurveda treatment in a black box design for the management of Rheumatoid arthritis- A community based study" (Sihmanad Guggulu) (under-THCRP)	1. Dr. Purnendu Panda, (CARI, Bhubaneswar)<br>2. Dr. Ashok Sinha, (RARI, Gangtok)<br>3. Dr. Anil Avhad,(RARI, Ahmedabad)<br>4. Dr. Monika Kumari, (RARI, Jaipur)<br>5. Dr. Shekhar , (RARI, Nagpur)		1. The Title of the project may be revised as "Effectiveness of **composite Ayurveda regimen** in a black box design for the management of Rheumatoid arthritis- A community based study".<br>2. In primary and secondary objectives the word 'complex Ayurveda treatment' may be replaced with 'composite Ayurveda regimen'.<br>**Recommendation:** Approved with the suggestion that the final proposal may be submitted incorporating the aforementioned suggestions to the CCRAS Headquarters for final approval.
3.2.12	"Effectiveness of complex Ayurveda treatment in a black box design for the management of Rheumatoid arthritis- A community based study" (Ayush SG) (under AMHCP-SCSP)	1. Dr. Seema Jain(CARI New Delhi)<br>2. Vd. Sandipkumar (CARI, Patiala)<br>3. Dr. Praveen Kumar K S(CARI Guwahati)<br>4. Dr. S.K Vedi (RARI,Jaipur)		1. The Title of the project may be revised as "Effectiveness of **composite Ayurveda regimen** in a black box design for the management of Rheumatoid arthritis- A community based study. (Ayush SG)".<br>2. In primary and secondary objectives the word 'complex Ayurveda treatment' may be replaced with 'composite Ayurveda regimen'.<br>**Recommendation:** Approved with the suggestion that the final proposal may be submitted incorporating the aforementioned suggestions to the CCRAS Headquarters for final approval.

		5. Dr. Amit Kumar (RARI,Gwalior)<br>6. Dr. Balaji potbhare (RARI,Patna)<br>7. DR. A. J. V. Sai Prasad (RARI,Vijaywada)<br>8. Dr. S. Asha (ALRARI, Chennai		
3.2.13	"Effectiveness of Rajahpravartani Vati and Saraswatarishta in Primary Dysmenorrhea-A prospective community based study" (under WCH-SCSP)	• Dr susmita P. otta, CARI, Bhubaneswar<br>• Dr. Rinku Tomar, CARI, Patiala<br>• Dr. Harit kumara, RARI, Lucknow<br>• Dr Kishor gavali, RARI, Jaipur<br>• Dr. vd. Priya Ashok Ras, RARI, Nagpur<br>• Dr Meghna PP, RARI, Trivandrum<br>• Dr Ritika Mishra, RARI, Patna<br>• Dr. Jeuti Rani Das, CARI, Guwahati<br>• Dr. Chris Antony, RARI, Mandi<br>• Dr. Jaiprakash, RARI, Ahmedabad		1. The Title of the project may be revised as "Effectiveness of Rajapravartani Vati and Saraswatarishta in **Dysmenorrhea**-A prospective community based study".<br>2. In primary and secondary objectives the word 'Primary Dysmenorrhea' may be replaced with 'Dysmenorrhea'.<br>3. Sample size of the project may be revised considering the prevalence of Dysmenorrhea to be 50%.<br>**Recommendation:** Approved with the suggestion that the final proposal may be submitted incorporating the aforementioned suggestions to the CCRAS Headquarters for final approval.

**Supplementary Agenda**				
	Study of the association of Prakriti with Gut microbiome/ metabolome and outcome of metastatic breast cancer	Dr. Manohar Gundeti, RO(Ay), CARI, Mumbai	Dr. Vikram Gota, Department of clinical pharmacology, ACTREC, TMC Mumbai	1. After discussion, the committee suggested to provide the soft copy of the proposal to the committee members for evaluation.<br>**Recommendation:** The decision of the experts will be communicated to the PI after receiving comments from experts.
**Agenda 3.3 Medicinal Plant Research**				
3.3.1	Identification of adulterants/substitutes for Ayurveda plant raw material in trade using pharmacognostic and phytochemical parameters.	**CARI, Jhansi**<br>1. Dr. Sanjeev Kumar Lale – PI and dealing officer<br>3. Mr. Ravi Chandra<br>2. Dr. Shidddamallayya, CCRAS, New Delhi	**NBRI, Lucknow**<br>1. Dr. Sharad Srivastava<br>2. Dr. K M Prabhu kumar;<br>3. Dr. Sanjeev Ojha	1. Ayurvedic plant name based monograph to be prepared.<br>2. The monograph to cover quality parameters of official, substitute and adulterants of the all studied botanical sources from both natural habitat and market.<br>**Recommendation:** Approved with the above suggestions. The final proposal may be submitted by incorporating the aforementioned suggestions to the CCRAS Headquarters for final approval.
3.3.2	A comparative study on hydroponic and field cultivated medicinal plants used in Ayurveda.	**CARI, Jhansi**<br>1. Dr. Jagdish Arya – PI and dealing officer<br>2. Dr. Shiddamallaya, CCRAS, New Delhi	**BHU, Varanasi**<br>1. Dr Shashi Pandey;<br>2. Dr S B Agarwal<br>3. Dr. Supriya Tiwari	1. Include the following plant species *Hemidesmus indicus* (L.) R.Br. ex Schult., *Desmodium gangeticum* (L.) DC. and *Rauvolfia serpentina* Benth. ex Kurz. in place of proposed study.<br>2. It is also suggested to study with previously proposed species on pilot basis, if found feasible can included in the project.<br>3. Revise the budget accordingly.<br>**Recommendation:** Approved with the above suggestions. The final proposal may be submitted by incorporating the aforementioned suggestions to the CCRAS Headquarters for final approval.
3.3.3	Comparative evaluation of field and hydroponic cultivation of selected Ayurvedic medicinal plants.	**CARI, Bengaluru 1.** Dr. V. Rama Rao – PI and dealing officer<br>2. Dr. Shiddamallaya, New Delhi	**ICAR, IIHR, Bengaluru**<br>1. Dr. K. Hima Bindu;<br>2. Dr. D. Kalaivannan<br>3. Dr Safeena	**Recommendation:** Approved
3.3.4	Study of seasonal variation on bioactive compounds of Panchavalkal-05 medicinal plants used in Ayurveda.	**CARI, Bengaluru**<br>**1.** Dr. V. Rama Rao – PI and dealing officer<br>2. Dr.	**GKVK, Bengaluru**<br>1. Dr. Srinivasappa, K. N<br>2. Dr. T.S. Manjunatha	1. It is suggested to collect Plant materials (bark from Primary stem) from same source in different seasons with GPS data.<br>2. Uniformity should be maintained while collecting the source plant material considering the location, maturity,

		Shiddamallaya, CCRAS New Delhi	Swamy	phenological growth, girth size of the plant etc.<br>3. Find out common phyto-constituents from Literature and select for the photochemical analysis.<br>**Recommendation:** Approved with the above suggestions. The final proposal may be submitted by incorporating the aforementioned suggestions to the CCRAS Headquarters for final approval.
3.3.5	*In vitro* propagation and genetic stability studies of *Ipomoea mauritiana* Jacq. -a Rare medicinal plant.	**NARIP, Cheruthuruthy**<br>1. Dr. Parvathy G Nair – PI and dealing officer<br>2. Ms. Thulasi, R<br>3. Dr. Shiddamallaya, CCRAS New Delhi	**Sree Neelakanta Government Sanskrit College, Pattambi, Palakkad, Kerala**<br>1. Dr. Vivek P. J.	**Recommendation:** Approved
3.3.6	Comprehensive inventory of Local Health Traditions (LHTs) & Ethnomedicinal Practices (EMPs) in India - Validation Through Ayurvedic and Ethnomedicinal Literure to Establish Uniqueness	**NIIMH, Hyderabad**<br>1. Dr. V. Sridevi<br>2. Dr. G.P. Prasad		1. Data collection should be taken from direct authentic website sources and also from available PG and Ph D thesis (like shodganga, inflibnet) on botany and anthropology and other related subjects.<br>2. One DEO/OA may be added in the man power.<br>3. Revise the budget accordingly.<br>**Recommendation:** Approved with the above suggestions. The final proposal may be submitted by incorporating the aforementioned suggestions to the CCRAS Headquarters for final approval.
3.3.7	Inventorization and Critical Appraisal of Medicinal Flora from Indigenous Literature of India.	**NIIMH, Hyderabad**<br>1. Dr. V. Sridevi -PI and dealing officer<br>2. Dr. G.P. Prasad<br>3. Dr. Shiddamallaya, CCRAS New Delhi<br>4. Dr. Bandi Venkateshwarlu,	**Tilak Maharashtra Vidyapeeth, Pune**<br>1. Dr. Manoja Abhijit Joshi	1. Timelines and deliverables of the project.<br>2. Scope of the project.<br>3. Preparation of a comprehensive data base and publication book<br>4. Model standard format for data presentation<br>5. Inclusion of one Co-PI from Literary Research section, CCRAS Hqrs, New Delhi.<br>6. Institutional over head charges<br>7. IPR issues<br>**Recommendation:** Approved the project proposal in principle. However a physical meeting to be convened by inviting PIs of the project to discuss the above issues.
3.3.8	Development of inventory of diet &	**NIIMH, Hyderabad**	**Tilak Maharashtr**	1. Timelines and deliverables of the project.

	dietetics from indigenous literature of India.	1. Dr. G.P. Prasad -PI and dealing officer<br>2. Dr. V. Sridevi<br>3. Dr. Bandi Venkateshwarlu, CCRAS New Delhi<br>4. Dr. Shiddamallayya	**a Vidyapeeth, Pune**<br>1. Dr. Abhijit H. Joshi<br>2. Dr. Manoja Abhijit Joshi	2. Scope of the project.<br>3. Preparation of a comprehensive data base and publication book<br>4. Model standard format for data presentation<br>5. Inclusion of one Co-PI from Literary Research section, CCRAS Hqrs, New Delhi.<br>6. Institutional over head charges<br>7. IPR issues<br>**Recommendation:** Approved the project proposal in principle. However a physical meeting to be convened by inviting PIs of the project to discuss the above issues.
3.3.9	Documentation of Traditional Food Recipes of Karnataka and their Validation with Ayurveda and Nutritional Perspective	**CARI, Bengaluru**<br>1. Dr. Sulochana Bhat - PI and dealing officer<br>2. Dr Shubhashree M.N<br>3. Dr. Bandi Venkateshwarlu, CCRAS New Delhi	**The University of Trans-Disciplinary Health Sciences and Technology, Bengaluru**<br>1. Dr. Megha;<br>2. Dr Subrahmanya Kumar	1. Uniqueness and difference in nature of study in comparison to the earlier studies of collaborative Institute.<br>2. Model standard format for data presentation<br>3. IPR issues<br>**Recommendation:** Approved the project proposal in principle. However a physical meeting to be convened by inviting PIs of the project to discuss the above issues.
3.3.10	Formulation and product development of Ayurveda recipes and nutritional evaluation of selected products.	**CARI, Bengaluru**<br>1. Dr. Sulochana Bhat- PI and dealing officer<br>2. Dr. Thejaswini;<br>3. Dr. Bhavya B.M;<br>4. Dr. Bandi Venkateshwarlu, CCRAS New Delhi	**CSIR-CFTRI, Mysore**<br>1. Dr. Sridevi Annapurna Singh<br>2. Dr. Ramaprasad TR<br>3. Dr. Prabhasankar<br>4. Dr. Chetana<br>5. Dr. Singam Reddy	**Recommendation:** Approved the project proposal in principle. However a physical meeting to be convened by inviting PIs of the project to discuss the study objective and deliverables.
**Agenda 3.4 Drug Standardization Research**				
3.4.1	Development of Quality standards of four medicinally important classical ayurvedic formulations.	Dr. Kousik Ghosh, RO(Chem), CSMCARI, Chennai		Quantitative markers analysis should be on dry weight basis. (µ/ml)<br>**Recommendation:** Approved
3.4.2	Quantitative estimation of	Dr. T. Vinod Kumar,		The committee suggested to add one Co-PI (Dr. Sujeet Mishra) from PCIM&H.

	phytochemicals and marker compounds in Kwath Churna samples and its decoction and their evaluation towards antimicrobial studies.	CSMCARI, Chennai		**Recommendation:** Approved with the suggestion that the final proposal may be submitted incorporating the aforementioned suggestions to the CCRAS Headquarters for final approval.
3.4.3	"Development of Quality Standards, Estimation of Markers and Shelf-Life Study of Chopchinyadi Churna, Marichadi Churna, Musali Churna & Saraswat Churna"	Mr. Puneet Kumar Singh, RO(Chem), CARI, Guwahati		**Recommendation:** Approved
**Supplementary Agenda**				
S1	Mechanistic studies in elucidation of *Shodhana* for nine poisonous medicinal plants of E1 schedule	1. Dr. Anagha Ranade<br>2. Dr. Vaibhav Chharde, CARI Jhansi (Co-Is)	1. Dr. Sharad Srivastava (PI)<br>2. Dr. Sanjeev Kumar Ojha (Co-I)	1. Council to take studies to assess the safety aspects of shodhita drugs in phase wise manner.<br>2. Accelerated shelf life study of the finished product needs to be added in the study.<br>3. Budgetary revision in terms of manpower may be made as per CCRAS research policy.<br>**Recommendation:** Approved with the suggestion that the final proposal may be submitted incorporating the aforementioned suggestions to the CCRAS Headquarters for final approval.
S2	Profiling of Phytochemicals in "Ayurvedic Medicinal Formulations" and Understanding their Aggregation Behavior	**1.** Dr. Vaibhav Chharde, CARI Jhansi<br>**2.** Dr Vijay Kumar, RO (Chemistry), Jhansi	1. Dr. Sairam Swaroop Mallajosyula, IIT Gandhinagar<br>2. Dr. Sriram Kanvah Gundimeda	1. Budgetary revisions are needed in contingency and manpower.<br>**Recommendation:** Approved with the suggestion that the final proposal may be submitted incorporating the aforementioned suggestions to the CCRAS Headquarters for final approval.
S3	Assessment of AYUSH-64 and Ayush Kwath to SARS-CoV-2 mutants on lung, liver and brain cells	Dr. A K Meena, RARI Gwalior	Dr. Hemchandra Jha, IT Indore	1. In Mile stones the work related to the following points only needs to be carried out:<br>**e.** In silico analysis of AYUSH-64, Ayush Kwath and their ingredients with SARS-CoV-2 proteins and

	and their organoids.			associated host factors at IIT Indore.<br>**f.** To use important wild types and mutants in SPIKE, E and Nucleocapsid proteins in the evaluation of the pathogenicity on Lung, Liver and Neural cells at IIT Indore.<br>**g.** Utilizes the two formulations to examine the affected host cells through in SARS-CoV-2 transfected proteins in Lung, Liver and Neural cells at IIT Indore.<br>**h.** Investigation of biomolecular changes upon SARS-CoV-2 proteins transfection and treatment with medicinal herbs through Raman spectroscopy at IIT Indore.<br>2. The study may be carried out in 501, Delta, Omicron variants of Covid.<br>3. The studies may be carried out in BSL-2 Laboratory.
S4	Aqueous and Hydroalcoholic extraction of selected Anukta Dravya plants and developing their various effective formulations for therapeutic uses		Dr. Pradeep Kumar Naik, Sambalpur University	1. Committee has suggested to incorporate only peculiar claims for further validation.<br>2. Efficacy studies to be carried out in phase 1 and based upon efficacy, toxicity study to be carried out.<br>**Recommendation:** Approved with the suggestion that the final proposal may be submitted incorporating the aforementioned suggestions to the CCRAS Headquarters for final approval.
**Agenda 3.5 Pharmaceutical Research**				
3.5.1	Process Validation and Pharmaceutical Standardization of Vacālaśunādi Taila, Somarājī Taila, Hiṅgutriguṇa Taila and Yastimadhu Ghrta	Dr. Suparna Saha, CARI, Kolkata		1. Furnitures (almira, file cabinet, etc.) and external storage devices may be removed from the budget.<br>**Recommendation:** Approved with the above suggestion. The final proposal may be submitted incorporating the aforementioned suggestion to the CCRAS Headquarters for final approval.
3.5.2	Development of quality standards for selected Arka formulations	Dr. Aswathi Mohan P, CSMCARI, Chennai		The committee suggested to share Arka preparation method with experts.<br>**Recommendation:** Approved
**Agenda 3.6 Pharmacology Research**				
3.6.1	Pre-clinical development of the identified herbs through AYUSH route for adjunct therapy in tuberculosis to reduce anti-tubercular treatment (ATT)	Dr. Vijay Kumar, Research Officer (Chemistry), Central Ayurveda Research Institute, Jhansi	Dr S K Rath Chief Scientist (Toxicology & Genotoxicity) Division of Toxicology	1. To carry out the efficacy studies first with respect to extracts of the test drug. Based on the leads toxicity studies may be initiated<br>2. Title may be changed as "Pre-clinical development of Amalaki (*Phyllanthus emblica* Linn.) fruit through AYUSH route as adjunct therapy in tuberculosis to reduce anti-tubercular treatment (ATT) induced hepatotoxicity"

	induced hepatotoxicity - Amalaki (*Phyllanthus emblica* Linn.)		& Experimental medicine CSIR-Central Drug Research Institute, Lucknow<br><br>Dr. Parvinder Pal Singh Principal Scientist (Natural Product & Medicinal Chemistry Division) CSIR-Indian Institute of Integrative Medicine, Jammu	**Recommendation:** Approved with the above suggestions. The final proposal may be submitted incorporating the aforementioned suggestions to the CCRAS Headquarters for final approval.
3.6.2	Pre-clinical development of the identified herbs through AYUSH route for adjunct therapy in tuberculosis to reduce anti-tubercular treatment (ATT) induced hepatotoxicity - Guduchi (*Tinospora cordifolia* (Thunb) Miers)	Dr. Vijay Kumar, Research Officer (Chemistry), Central Ayurveda Research Institute, Jhansi	Dr. Virendra kumar Maheshbhai Prajapati Senior Scientist (Toxicology & Pathology) Division of Toxicology & Experimental medicine CSIR-Central Drug Research Institute, Lucknow<br><br>Dr Rashmi Sharma Scientist (Infectious Diseases Division, Microbiology) CSIR-Indian Institute of Integrative	1. To carry out the efficacy studies first with respect to extracts of the test drug. Based on the leads toxicity studies may be initiated<br>2. Title may be changed as "Pre-clinical development of Guduchi (*Tinospora cordifolia* (Thunb) Miers) stem through AYUSH route as adjunct therapy in tuberculosis to reduce anti-tubercular treatment (ATT) induced hepatotoxicity"<br>**Recommendation:** Approved with the above suggestions. The final proposal may be submitted incorporating the aforementioned suggestions to the CCRAS Headquarters for final approval.

			Medicine, Jammu	
3.6.3	Pre-clinical development of the identified herbs through AYUSH route for adjunct therapy in tuberculosis to reduce anti-tubercular treatment (ATT) induced hepatotoxicity - Vasa (*Justicia adhatoda* Linn.)	Dr. Vijay Kumar, Research Officer (Chemistry), Central Ayurveda Research Institute, Jhansi	Dr. Virendra kumar Maheshbhai Prajapati Senior Scientist (Toxicology & Pathology) CSIR-Central Drug Research Institute, Lucknow<br><br>Dr. Utpal Nandi Sr. Scientist (Pharmacokinetics) CSIR-Indian Institute of Integrative Medicine, Jammu	1. It was suggested to carry out the efficacy studies first with respect to extracts of the test drug. Based on the leads toxicity studies may be initiated<br>2. Title may be changed as "Pre-clinical development of Vasa (*Justicia adhatoda* Linn.) leaves through AYUSH route as adjunct therapy in tuberculosis to reduce anti-tubercular treatment (ATT) induced hepatotoxicity<br>**Recommendation:** Approved with the above suggestions. The final proposal may be submitted incorporating the aforementioned suggestions to the CCRAS Headquarters for final approval.
3.6.4	Preclinical study of Dhatri lauha (A classical Ayurvedic formulation) in iron deficiency anemia (Panduroga)	Dr. Vaibhav Charde Research Officer (Ayurveda) Central Ayurveda Research Institute, Jhansi	Dr S K Rath Chief Scientist (Toxicology & Genotoxicity) CSIR-Central Drug Research Institute, Lucknow	1. The proposed protocols for toxicity studies were approved by the Committee.<br>2. The title of the project may be modified as 'Preclinical toxicity study of Dhatri lauha'.<br>3. It was suggested to use honey as Anupana (vehicle) for the administration of test drug as mentioned in Ayurvedic Formulary of India (AFI).<br>**Recommendation:** Approved with the above suggestions. The final proposal may be submitted incorporating the aforementioned suggestions to the CCRAS Headquarters for final approval.
3.6.5	Development of Mahua (*Madhuca longifolia*) based food recipe (Product development) and its nutritional, toxicity, efficacy and bioavailability studies	Dr. Goli Penchala Prasad, Assistant Director In-charge National Institute of Indian Medical Heritage, Hyderabad	Dr. J Sreenivasa Rao, Scientist E/Deputy Director ICMR-National Institute of Nutrition-Hyderabad	1. To carry out the efficacy studies first with respect to test drug before initiating toxicity studies.<br>2. The recipe forms being formulated/ tested may be mentioned.<br>3. Efficacy studies to be carried out in the animal models of nutrition deficiency anemia also.<br>4. The title of the project may be modified as 'Development of *Madhuca longifolia* (Flower) based food recipe (Product development) and its nutritional, toxicity, efficacy and bioavailability

				studies'<br>5. The manpower in the project was restricted to SRF (One No.), JRF (one No.) and MTS (1 No.).<br>6. Provision of laptop is not allowed.<br>**Recommendation:** Approved with the above suggestions. The final proposal may be submitted incorporating the aforementioned suggestions to the CCRAS Headquarters for final approval.
3.6.6	Development of *Opuntia elatior* (fruit) based food recipes (product development) and its nutritional, toxicity, efficacy and bioavailability studies.	Dr.V. Sridevi M.D.(Ayurveda) Research Officer (Ayurveda) National Institute of Indian Medical Heritage, Hyderabad	Dr. J Sreenivasa Rao, Scientist E/Deputy Director ICMR-National Institute of Nutrition-Hyderabad	1. To carry out the efficacy studies first with respect to test drug before initiating toxicity studies.<br>2. The recipe forms being formulated and screened may be mentioned.<br>3. Efficacy studies to be carried out in the animal models of nutrition deficiency anemia also.<br>4. The manpower in the project was restricted to SRF (Two Nos.) and Project Assistant (1 No.).<br>5. Provision of laptop is not allowed.<br>**Recommendation:** Approved with the above suggestions. The final proposal may be submitted incorporating the aforementioned suggestions to the CCRAS Headquarters for final approval.
3.6.7	Evaluation of anti-cancerous potential, *in silico* pharmacological analysis and molecular mechanism of *Kanchanar guggulu* in ovarian cancer µTumor spheroids, and in *in vivo* models.	Dr. Amit Kumar Dixit Assistant Director (Bio-Chemistry), Central Ayurveda Research Institute, Kolkata	Dr. A.K. Srivastava, Scientist, Indian Institute for Chemical Biology-CSIR Kolkata	1. To carry out combinations studies of test drug and standard drug to establish the efficacy of test drug in reducing the dose of standard drug in treatment of cancer.<br>2. A systematic study has to be executed so as to establish the efficacy of test drug for further clinical use.<br>3. Progress of the project with respect to objectives and outcomes are to be monitored from subject expert (Oncology) at periodic intervals.<br>**Recommendation:** Approved with the above suggestions. The final proposal may be submitted incorporating the aforementioned suggestions to the CCRAS Headquarters for final approval.
3.6.8	Evaluation of Protective Effect of *Punarnavadi kwatha churna* on Adenine-Induced Chronic Kidney Disease in Wistar rats.	Dr. Manajit Bora Research Officer (Pharmacology) Central Ayurveda Research Institute, Guwahati	Dr. Pritam Mohan College of Veterinary Science, Assam Agricultural University, Khanapara, Guwahati	**Recommendation:** The project proposal was deferred.
3.6.9	Biochemical Effect of Trikatu churna	Dr.N. Thamizh	Not Applicable	1. Outcome of the study should aim towards management of subclinical

	in Methimazole induced Experimental Hypothyroidism in Wistar Rats	Selvam Assistant Director (Biochemistry), National Ayurveda Research Institute for Panchakarma, Cheruthuruthy, Thrissur		hypothyroidism.<br>2. Suitable Biochemical parameters may be included.<br>3. Combination studies with test drug and standard drug may also be considered.<br>4. Budget may be revised accordingly.<br>**Recommendation:** Approved with the above suggestions. The final proposal may be submitted incorporating the aforementioned suggestions to the CCRAS Headquarters for final approval.
3.6.10	Toxicity profile of Bhasma/ rasakalpa Rasamanikya Rasa in experimental animals	Dr. Lalrin Puia Research Officer (Pharmacology) Central Ayurveda Research Institute-Kolkata	Dr AB Pant Senior Principal Scientist & Professor (AcSIR), CSIR-Indian Institute of Toxicology Research Lucknow, India	1. The proposed protocols for toxicity studies were approved by the committee.<br>2. Title to be modified as "Toxicity profile of Rasamanikya Rasa in experimental animals".<br>3. It was suggested to use honey as anupana (vehicle) for the administration of test drug as mentioned in Ayurvedic Formulary of India (AFI).<br>**Recommendation:** Approved with the above suggestions. The final proposal may be submitted incorporating the aforementioned suggestions to the CCRAS Headquarters for final approval.
3.6.11	Toxicity profile of Bhasma/ rasakalpa Tribhuvana kirti Rasa in experimental animals	Dr. Aruna Devi Research Officer (Pharmacology) CSM Central Ayurveda Research Institute - Chennai Tamilnadu	Dr AB Pant Senior Principal Scientist & Professor (AcSIR), CSIR-Indian Institute of Toxicology Research Lucknow, India	1. The proposed protocols for toxicity studies were approved by the committee.<br>2. Title to be modified as "Toxicity Profile of Tribhuvana kirti Rasa in experimental animals".<br>3. It was suggested to use honey as anupana (vehicle) for the administration of test drug as mentioned in Ayurvedic Formulary of India (AFI).<br>**Recommendation:** Approved with the above suggestions. The final proposal may be submitted incorporating the aforementioned suggestions to the CCRAS Headquarters for final approval.
3.6.12	Toxicity profile of Bhasma/ rasakalpa Swaskuthar Rasa in experimental animals	Dr. Srikanth Ala Research officer (Pharmacology) National Ayurveda Research Institute for Panchakarma, Cheruthuruthy, Thrissur Cheruthuruthy	Dr Dhirendra Singh Senior Principal Scientist CSIR-Indian Institute of Toxicology Research Lucknow, (UP), India	1. The proposed protocols for toxicity studies were approved by the committee.<br>2. Title to be modified as "Toxicity profile of Swaskuthar Rasa in experimental animals".<br>3. It was suggested to use honey as anupana (vehicle) for the administration of test drug as mentioned in Ayurvedic Formulary of India (AFI).<br>**Recommendation:** Approved with the above suggestions. The final proposal may be submitted incorporating the aforementioned suggestions to the CCRAS Headquarters for final approval.

		, Thrissur, Kerala		
3.6.13	Toxicity profile of Bhasma/ rasakalpa Mrityunjaya Rasa in experimental animals	Dr. Allakonda Lingesh Research Officer (Pharmacology) Regional Ayurveda Research Institute- Gwalior	Dr Dhirendra Singh Senior Principal Scientist CSIR-Indian Institute of Toxicology Research Lucknow, (UP), India	1. The proposed protocols for toxicity studies were approved by the committee.<br>2. Title to be modified as "Toxicity profile of Mrityunjaya Rasa in experimental animals".<br>3. It was suggested to use honey as Anupana (vehicle) for the administration of test drug as mentioned in Ayurvedic Formulary of India (AFI).<br>**Recommendation:** Approved with the above suggestions. The final proposal may be submitted incorporating the aforementioned suggestions to the CCRAS Headquarters for final approval.
3.6.14	Preclinical study on efficacy, safety and toxicity of Swarna Prashan Regimen as Adjunct therapy in pediatric acute lymphoblastic leukemia.	Dr. Kuldeep Chaudhary Research Officer (Ayurveda) RRAP-Central Ayurveda Research Institute-Mumbai	Dr. Vikas Dighe Scientist 'E', ICMR - National Institute for Research in reproductive and Child Health-Mumbai.	1. The solubility of test drug should be ascertained while carrying out in vitro cell line studies.<br>2. To carry out combinations studies of test drug and standard drug to establish the efficacy of test drug in reducing the dose of standard drug in treatment of cancer.<br>3. A systematic study has to be executed so as to establish the efficacy of test drug for further clinical use.<br>4. Progress of the project with respect to objectives and outcomes are to be monitored from subject expert (Oncology) at periodic intervals.<br>**Recommendation:** Approved with the above suggestions. The final proposal may be submitted incorporating the aforementioned suggestions to the CCRAS Headquarters for final approval.
**Agenda 3.7 Literary and Fundamental Research**				
3.7.1	Transliteration & translation of *Vakyapradeepika* commentary of *Ashtangahridaya*	Dr. V.K. Lavaniya, R.O. (Ayu.)	Prof. K. Murali, Arya Vaidya Sala, Kottakal	Duration of the project may be kept as one year.<br>**Recommendation:** Approved with the above suggestion. The final proposal may be submitted incorporating the aforementioned suggestion to the CCRAS Headquarters for final approval.
3.7.2	*Mahāmati CakrapāṇiDāsa Kṛta Abhinava Cintāmaṇi* (Sanskrit to English translation)	Dr. V. Sridevi	Not Applicable	**Recommendation:** Approved
3.7.3	Transcription and critical edition of various manuscripts of *Sata KanthaRatnaNigha*	Dr. Satyabrata Nanda, R.A (Sanskrit)	Not Appli\cable	Duration of the project may be kept as one year with one SRF Ayurveda/Sanskrit.<br>**Recommendation:** Approved with the above suggestion. The final proposal may be submitted incorporating the aforementioned suggestion to the CCRAS Headquarters for

	ntu. (A Palm Leaf Manuscript)			final approval.
3.7.4	Insilico – network Pharmacology study of select decoction (*Kashaya*) from national list of essential Ayush medicines (NLEAM) – 2022 and Ayush 64	-	Dr. Uddhavesh Sonawane, CDAC Pune	1. Title may be changed to "Insilico network pharmacology study of selected coded formulations of CCRAS".<br>2. Formulations may be finalized in consultation with CCRAS Drug Cell.<br>**Recommendation:** Approved with the above suggestion. The final proposal may be submitted incorporating the aforementioned suggestion to the CCRAS Headquarters for final approval.
